# Data collection methods in qualitative research: researchers' reflections

**DOI:** 10.3389/frma.2026.1778160

**Published:** 2026-02-23

**Authors:** Bharat Prasad Neupane, Niroj Dahal, Rebat Kumar Dhakal, Md. Kamrul Hasan, Joseph A. Villarama, Bench Go Fabros

**Affiliations:** 1 Kathmandu University School of Education, Hattiban, Lalitpur, Nepal; 2 Department of English, United International University, Dhaka, Bangladesh; 3 Central Luzon State University, Nueva Ecija, Philippines

**Keywords:** qualitative research, interview, observation, focus group discussion, self-reflection, innovative data collection methods

## Abstract

This article explores qualitative data collection methods based on researchers' experiences, integrating established techniques with culturally situated innovations. It explores core methods—interviews, observations, focus group discussions, and self-reflection—featuring their applications, strengths, and challenges in capturing nuanced human experiences. Moving beyond conventional frameworks, the article introduces and elaborates on innovative, context-sensitive data collection approaches such as *Kurakani* (informal conversational inquiry), *Pandheri Guff* (contemplative dialogue among women), and *Chautari Guff* (public participatory dialogue). Grounded in the authors' critical reflections from their own research journeys, the discussion offers practical insights for both novice and experienced researchers. It advocates methodological creativity and epistemic diversity, arguing that thoughtful adaptation and the generation of methods aligned with local contexts enhance rigor, authenticity, and the ethical co-construction of knowledge in qualitative research.

## 1 Introduction

Qualitative research is a unique and complex approach to understanding the phenomenon. Unlike quantitative research, which relies on numbers and statistics to understand facts through reductionist approaches, qualitative research explores the intricacies of complex problems, phenomena, experiences, behaviors, and the affective dimensions of human experiences. When a researcher needs to investigate a research problem and is unsure of the variables, qualitative research is the most appropriate method (Creswell, 2014). Qualitative research is like exploring a complex puzzle, where every piece is a story waiting to be told or unfolded. Because of this, as (Denzin and Lincoln 2018) argue, it is essential to capture the richness of subjective realities. Qualitative research enables researchers to understand the “how” and “why” behind human behavior, rather than just the “what”. (Denzin and Lincoln 2018) emphasize that qualitative methods are essential for addressing complex questions that quantitative methods alone cannot answer. In qualitative research, a central phenomenon is the main idea, topic, or procedure under examination or exploration. Thus, to gain a deeper understanding of the social phenomenon, qualitative research is helpful, as it reinstates participants' active role, transforming them into cognizing subjects (Merriam and Tisdell, 2016).

The typical characteristics of the quantitative paradigm relate to the methods of observation that researchers generally employ to prove, validate, or establish the cause-and-effect relationship of the phenomenon (Bonilla-Medina and Samac'a-Boh'orquez, 2023; Merriam and Tisdell, 2016). While quantitative research attempts to capture the objective reality of the phenomenon with generalizability as the key characteristic of its outcome, the ideas such as relativity, uncertainty, particularity, and subjectivity that qualitative research upholds are closely related to the thought that reality is socially constructed, aligned with relational onto-epistemo-axiological orientations. This implies that there are various views of what truth, knowledge, and value are, rather than a singular, unchanging method of perceiving and comprehending the world (Merriam and Tisdell, 2016). These paradigms are all categorized under the umbrella of qualitative research. In addition to the ongoing quest for reality, knowledge, and value, qualitative research plays a significant role for educational researchers at various phases of their career development, particularly for those who have completed undergraduate and graduate studies (Castañeda-Trujillo and Losada-Rivas, 2024). Quantitative methods shall use closed-ended techniques, where the researcher provides predetermined response categories (such as “strongly disagree,” “disagree,” “agree,” and “strongly agree”). In contrast, qualitative research employs more open-ended methods, allowing participants to help shape possible answers to the researcher's general questions. This method involves more than just answering questions; it involves participants as co-constructors of knowledge.

Some of the research designs commonly associated with qualitative research include autoethnography, ethnography, narrative inquiry, case study, phenomenology, participatory action research, and grounded theory, to name but a few that employ different data collection techniques or approaches. Autoethnography uses the researcher's lived experiences—through journaling, memory work, self-observation, and personal artifacts blended with qualitative tools like interviews and participant observation—as primary data to reflexively interpret the self within broader cultural, political, and social contexts, blurring the boundaries between data collection and analysis while treating writing itself as a process of inquiry (Dahal, 2025b; Dahal and Luitel, 2022). Ethnographic designs are qualitative methods for characterizing, evaluating, and understanding the common patterns of language, behavior, and beliefs that emerge over time within a cultural group (Creswell, 2014). In ethnography, the researcher employs a range of information collection methods, including observation, field notes, and interviews, to create a comprehensive picture of the culture-sharing community.

At times, researchers might be interested in sharing the tales of one or two people rather than a culture-sharing community. In such cases, researchers employ narrative research designs to compose narratives about people's experiences, explain their lives, and gather and share stories about those individuals (Riessman, 2008). The participants' stories are co-constructed through interviews and informal conversations. The researchers may blend their lived experiences with participants' stories (Clandinin and Connelly, 2000). A case study is mainly conducted for an in-depth study of an organization (s), individual (s), problem (s), or event (s) from multiple perspectives. In case study design, researchers employ a range of data-gathering techniques, such as interviews, informal conversations, fieldwork, and focus group discussions, to gather comprehensive information over an extended period (Stake, 1995; Yin, 2009, 2012), involving different stakeholders, for a comprehensive understanding of the chosen case (s) for its contextual understanding. Whereas in phenomenology, a method of inquiry originating in philosophy and psychology, the researcher describes participants‘ lived experiences with a phenomenon. For multiple people who have all witnessed the occurrence, this description captures the essence of their experiences through a prolonged period of open and unstructured interviews and informal conversations (Giorgi, 2009; Moustakas, 1994). With its roots in democracy and social justice, participatory action research places a strong emphasis on coordinated inquiry to effect social change (Smit et al., 2024). In educational settings, PAR engages co-researchers as key stakeholders who, together with researchers, identify problems, plan and implement actions, observe outcomes, and reflect on generating solutions. In PAR, data collection emphasizes collaboration and co-creation. Methods such as focus groups, interviews, mapping, photovoice, participant observation, and community-led tools are used to ensure that participants actively shape both the process and the outcomes. Grounded theory designs are methodological, qualitative techniques used to produce a comprehensive explanation of a process, activity, or interpersonal interaction and to gain a general understanding based on participants' perspectives and experiences (Charmaz, 2006; Corbin and Strauss, 2007). Different qualitative data collection processes, ranging from semi-structured interviews to informal conversations, are relevant methods in grounded theory. The main steps in creating this theory are gathering interview data, classifying and connecting information into categories (or themes), and creating a figure or visual model that illustrates the whole explanation. The elucidation is thus “grounded” in the participant data. Therefore, eliciting quality and in-depth data until saturation is not achieved to develop a theory is crucial.

Indeed, qualitative approaches employ a variety of designs, have distinct data collection and analysis stages, and rely on textual and visual data. Setting the study parameters, gathering data via semi-structured or (un)structured interviews and observations, visual materials and documents, and creating a procedure to elicit information are all steps in the data collection process (Creswell, 2014). Researchers face several challenges during data collection. When observing research participants, the researchers may be perceived as intrusive. Additionally, during interviews with research participants, the researchers‘ presence may prompt biased responses. While collecting and documenting data, the researchers may find that not everyone is equally perceptive or articulate. In this context, this article aims to examine the nuances of various data collection methods and articulate the unique contributions of qualitative methods, emphasizing their ability to address questions of meaning, context, and human experience through the experiences of Nepali researchers. From the brief discussion of the nuances of different qualitative research designs and data collection methods, it becomes evident that, regardless of the design employed, it demands a deep dive into participants' lived experiences, beliefs, values, perceptions, and emotions. This involves employing a range of methods, including (un)structured and semi-structured interviews, participant and non-participant observations, focus group discussions, self-reflection, and innovative methods. Each method is carefully chosen for its practicality and alignment with the research agenda, question(s), and the chosen method. It is like selecting the right tool for the right job. These methods help researchers connect with people on a deeper level and gain insights that might not be possible with other approaches.

Thus, this article seeks to feature data collection approaches across different qualitative research designs, thereby advocating their broader adoption and integration into mainstream research practices. Although this article does not have a traditional, distinct methods section, it integrates methodological discussions throughout its exploration of data collection techniques and/or approaches. Drawing on (Denzin and Lincoln 2018) framework, this article critically engages with data collection methods, including interviews and informal conversations, observations, focus group discussions, and self-reflection. Besides, the study also elucidates a few innovative or emerging data collection methods based on the South Asian context in general and Nepal in particular, such as “*Kurakani”*, “*Pandheri Guff* ”, *and “Chautari Guff”*. *Kurakani* is an informal conversational inquiry that builds authentic trust through casual dialogue, moving beyond rigid Western interview structures. *Pandheri Guff* is a contemplative dialogue in safe spaces (evening/morning conversations among women) that captures narratives that might remain hidden in formal settings. *Chautari Guff* is illustrated as a public participatory dialogue at traditional community gathering places, emphasizing the co-construction of collective knowledge. These approaches offer the space for the commitment to blending theoretical reflection with practical application.

## 2 Interview and informal conversation

Interviews are the primary method of data collection across various qualitative research designs. Qualitative researchers mainly conduct interviews to elicit participants‘ perspectives, allowing them in-depth and nuanced insights into their perceptions and experiences. The interview enables researchers to collect in-depth insights into participants' beliefs, values, emotions, and experiences through in-person conversation. The qualitative interview is less structured, with participants who actively contribute to meaning-making rather than respondents from whom data are elicited (DiCicco-Bloom and Crabtree, 2006). Although there are primarily three ways of conducting interviews, namely structured, semi-structured, and unstructured, unstructured and semi-structured are the commonly adopted formats in qualitative research, as these are flexible and encourage participants to explore the phenomena or issues under investigation more deeply. As the most common format in qualitative research, semi-structured interviews blend the features of structured (set questions) and unstructured (flexible participant expression).

Researchers prepare questions for the interviews and simultaneously enable participants to have a natural conversation. In unstructured interviews, there may not be a fixed set of guiding questions, but researchers always keep the thematic areas at the back of their mind during the conversation. In contrast, a carefully designed set of questions guides the structured interviews. An interview should not be haphazard, no matter the type of interview we conduct. Hence, careful planning and applying the process of conducting interviews is crucial. The interview begins by crafting practical and effective questions or strategies to elicit in-depth information on the investigation issue. However, simply asking the carefully designed interview question is not enough. Most novice researchers face the challenge of not being able to collect enough data. The problem arises if they are unable to ask the right probing questions in between. Questions like “Could you please elaborate further?” Could you please give an example to illustrate what you claimed? and “May I get a bit more detail on this?” and/or similar questions may help researchers get in-depth information about the issue. Besides, obtaining prior consent to record and transcribe the interview for accurate documentation is equally crucial for capturing the participants' voices (Bell et al., 2022).

As researchers develop close connections with participants, they may inadvertently intrude into the private spheres of the participants, and at times, they can elicit confessional responses. Therefore, researchers must be cautious and maintain trust and respect for their participants‘ experiences (Balasanyan and Gevorgyan, 2024). Additionally, adapting to the language styles according to the participants' cultural backgrounds may enhance rapport and enable researchers to collect authentic, high-quality, and in-depth data (Elhami et al., 2024). Qualitative interviewing is a robust method for understanding intricate human experiences. Nevertheless, it is time-consuming and can influence researchers' bias during data collection. Likewise, it is also challenging, and researchers must carefully consider ethical issues that may arise during the interview. Considering ethical issues is essential in qualitative research. Different interview methods may be suitable for various research designs. Even semi-structured and unstructured methods are the most common qualitative data collection approaches. While unstructured and semi-structured interviews are common in phenomenology and narrative inquiry for collecting participants' experiences through in-depth interviews, semi-structured interviews might suffice in case studies, grounded theory, and, to some extent, even ethnography.

In this section, I, the first author, reflect on how I employed interviews in narrative research during my PhD study (Neupane, 2023). As I subscribed to the life-history approach to narrative inquiry as a research method, four participants‘ lived stories were developed by conducting in-depth interviews (Kvale and Brinkmann, 2009), a most common data collection method in language teaching and learning research (Barkhuizen et al., 2014). The purpose of my study was to explore the lived experiences of secondary-level English-language teachers and to examine the trajectory of identity negotiation through life history. The research was guided by the overarching research question: What is the trajectory of identity negotiation of secondary-level English language teachers from community schools in Nepal? This question was pertinent for understanding how secondary-level English language teachers from public schools in Nepal negotiated their identities. My study addressed three subsidiary research questions: (1) How do beliefs and emotions shape the identity of secondary-level English language teachers? (2) How does imagined identity contribute to the identity development of English language teachers? and (3) How do sociocultural environments and agency influence the identity negotiation of English language teachers? First, I collected participants' stories through unstructured conversations to elicit accounts of their various life episodes, including childhood, basic education, secondary education, university education, and teaching experience. During the first phase of story generation, I kept the thematic headings and their life episodes in mind. I did not use any preset questions during this stage. However, open-ended, semi-structured questions were asked afterward to elicit stories during the story generation process, as (Hollway and Jefferson 2000) stated. For further details, participants were asked to provide additional information and include relevant examples of incidents. Along with the major preset questions (Richards, 2003), follow-up probing questions relating to specific incidents were asked to elicit further details about the incident. As noted by (Savin-Baden and Niekerk 2007), I patiently listened to the participants‘ stories during the interview process. I actively participated in the story generation, acknowledging the mutually co-constructed nature of the stories (Sfard and Prusak, 2005). As experiences are recorded and shared, interviews and informal conversations remain the most effective tools for eliciting participants' experiences.

While conversing, I was mindful not to distract participants from the flow of their storytelling, though I sometimes asked follow-up questions to dig out more essential details and examples of some of the crucial incidents. Thus, the participants and I co-constructed the life stories (Kvale and Brinkmann, 2009) of the learning experiences of these four English language teachers. I recorded the interview and informal conversation with the participants' consent. And even those oral consents were recorded. After that, I transcribed and translated the data for further interpretation and meaning-making.

## 3 Observation and *kurakani*

In conducting qualitative research, particularly in ethnographic fieldwork, the concepts of observation and *Kurakani* [informal conversations] play pivotal roles in shaping the research experience and outcomes. Before delving into the processes of fieldwork, it is essential to situate the research within the broader context of ethnographic inquiry. The purpose of my study was to explore the understanding and practices of gender inclusion in school governance in a rural Nepali community school setting. To address the purpose, I devised three research questions: (1) How do SMC members understand “inclusive school governance” in a rural community school in Nepal?, (2) How do women stakeholders get into the School Management Committee in a rural community school setting? and (3) How do women SMC members negotiate their participation in school decision-making? Throughout my PhD study (Dhakal, 2021), I, the third author, regarded all interactions—whether formal or informal—as integral to my fieldwork. This perspective allowed me to engage with participants in diverse settings, including their homes, schools, and community spaces. For instance, I visited participants at various locations, including Lakeside in Pokhara, a hotel in Kathmandu, and my flat in Bhaktapur, Nepal. These interactions extended beyond mere observation; they included regular telephone follow-ups that contributed significantly to my understanding of the participants' perspectives.

The fieldwork unfolded in three major phases, each emphasizing the importance of observation and *Kurakani* (informal conversation). The first phase was ‘familiarization', during that phase, I immersed myself in the school community. My primary objective was to understand the dynamics of the school environment and establish rapport with key informants, including members of the School Management Committee (SMC). This phase involved observing informal interactions among teachers and engaging in casual conversations that revealed insights into their roles and relationships within the school. My participation in activities like serving on a teacher selection committee further solidified my position within this community, allowing me to transition from an outsider to an insider (Dhakal, 2019) - a full member of their school. I also learnt that, unlike my theoretical idea of being a “fully observe” or “external researcher”, my positionality was constantly under negotiation in the field. Upon returning from the field, I reflected that my ‘participant-as-observer' (Gold, 1958) role enabled me to fully understand the study's context and alleviated potential hesitations and mistrust on the part of the research participants toward me. One advantage of participating in the daily life of people was that it also enabled the researcher to “get a deeper insight into the culture of the people being studied” (Takyi, 2015; p. 864). However, I also sensed that I was going too native (Madden, 2017) – nearly losing my original purpose and identity as a researcher. This reflection taught me to balance my role as ‘observer as participant' and ‘participant as observer' as per the context.

The second phase largely comprised ‘observation and initial engagement' in which I adopted a more observational stance while also engaging in *Kurakani* with participants. In this phase, I initially became a complete observer (Gold, 1958; Takyi, 2015), as the discussion of forming a new SMC was a hot topic in school but a subject of little concern in the community. As such, I observed some informal discussions among the teachers at school and also heard about partisan lobbying for the Chairperson's position. Moreover, the SMC members requested that I also facilitate a SWOT analysis of the school and assist in developing the strategic plan. Although I initially frowned upon the idea of engaging at such a deeper level in their meeting, the event set a positive tone for the interaction with the SMC members. This event made my subsequent meetings and interactions with the women SMC members more engaging and participatory – they showed their interest in their Kurakani as much as I was in talking to them. This further helped me utilize *Kurakani* (informal talks, chitchats, or conversational style of dialogue) as a method of ethnographic inquiry, a concept (Desjarlais 2003) termed *Kuragraphy*, which (Rai 2013), and (Meche 2019) also followed. Since my approach to engaging with participants was informal and conversational, I did not use predefined research tools, such as ‘*Kurakani questions*' or ‘group interaction guidelines'; however, the research questions guided me throughout. The discussions surrounding the formation of a new SMC provided a rich backdrop for informal dialogues. I observed informal discussions among teachers and community members that illuminated the political landscape shaping SMC dynamics. Attending key gatherings not only allowed me to witness decision-making processes but also facilitated deeper connections through informal conversations. For example, a participant's invitation to her home fostered a familial bond that enhanced their interactions.

Such moments exemplified how *Kurakani* enriched my understanding of their social fabric. The final phase involved “extensive engagement” with participants through *Kurakani* and reflective practices. I conducted individual and group discussions that were guided by informal dialogue rather than rigid research tools. This conversational approach enabled me to clarify participants' statements and collaboratively construct meanings from their expressions. The absence of structured questions allowed for a more organic flow of conversation, fostering trust and openness among participants. Overall, observation and *Kurakani* are fundamental components of qualitative ethnographic research that facilitate deeper insights into cultural contexts. By embracing informal dialogues alongside structured observations, researchers can cultivate richer relationships with participants and gain more nuanced understandings of their lived experiences. This approach enhances data collection as well as transforms the researcher-participant dynamic into a collaborative exploration of knowledge.

## 4 Focus group discussion

Like interviews and observations, focus group discussions (FGD) are widely employed data collection methods in qualitative research, allowing for the exploration of complex social issues, behaviors, and attitudes within a group through interaction. FGD is appropriate when researchers aim to explore participants' collective insights grounded in a specific sociocultural context, which interviews, observations, and surveys fail to capture (Krueger and Casey, 2009). In this section, I, the first author, share my experience of conducting FGD along with the theoretical underpinnings, methodological implications, possibilities, and challenges of integrating it in qualitative research to collect data.

In general, focus group discussions are conducted in a structured format, with the researcher moderating the dialogue among 6 to 12 participants on the issue being explored (Morgan, 1997). Unlike an interview, FGD emphasizes the group dynamics where participants develop and refine their concepts, ideas, perceptions, and understandings through interactions with other members, challenge their own and others‘ assumptions, and co-construct shared meanings. We mainly conduct FGD to explore how and why participants experience, interpret, and understand particular phenomena (Barbour, 2007). FGD is primarily conducted to understand the issue for further research, to evaluate educational programs, policies, and practices, to explore and understand complex behaviors, and to identify the contextual and cultural influences on decision-making (Stewart et al., 2007). During the context, input, process, and product (CIPP) evaluation of MEd in English Language Education curriculum, I conducted a focus group in order to collect students' feedback on the curriculum, pedagogy, assessment, and other issues related to the course. For that, I carefully selected participants based on commonalities in key characteristics (students who studied the same curriculum in different batches). However, I was also aware of avoiding extreme homogeneity as it could hamper diverse opinions. Hence, maintaining a balance between homogeneity and divergence through purposive selection is critical (Kitzinger, 1995). During the FGD my role as a researcher was crucial for generating ideas on the phenomenon and resolving conflicting viewpoints and problems that may arise during the discussion. For smooth conduction of the FGD, first I built rapport, developed norms, and encouraged equal participation and voice among participants, asking probing questions to elicit details and reduce ambiguities, and managing emotional situations (Rabiee, 2004). During times of confusion or ambiguity, I asked questions such as, “Could you please elaborate on the point further?” or “Could you please provide an example of how you did that particular task?” I used semi-structured interviews in focus group discussions, providing a flexible environment for developing themes. I conducted FGD in a neutral venue that was equally accessible to all participants for their ease. FGD sessions lasted 60–90 min and were audio- and video-recorded, with participants‘ prior consent for the recordings' accuracy and precision. During the FGD, I took note of body language and non-verbal cues that provide contextual background for the transcript (Wilkinson, 1998).

FGD enables researchers to elicit layered data as participants interact, react to, refine, and counterargue one another's viewpoints on the issue of exploration. This sort of dialogic environment encourages participants to express their perspectives, which would otherwise remain unexpressed in one-to-one interviews and informal conversations (Morgan, 1997). FGDs are relatively time- and cost-effective compared with interviews, informal conversations, and surveys (Bloor et al., 2001). Despite many merits, if the moderators are unable to balance power during interaction, assertive and outspoken participants may overshadow quieter members. In addition, the tendency to groupthink – a practice of confirming the majority voices may hinder and suppress the unique perspectives (Kitzinger, 1995). In such cases, moderators should be cautious and ask direct questions, such as, “What do other participants think about this?” Please share your divergent opinions freely. Thus, to make the FGD effective, one may conduct a mock FGD or pilot testing to refine the questions and to identify and prepare the logistics (Stewart et al., 2007). Member checking can be conducted after the discussion and interpretation are shared with participants (Barbour, 2007). Thus, FGD remains a pivotal method to collect data in qualitative research. Though challenges such as moderator bias persist, compliance with methodological procedures to maintain rigor and ethical standards enables researchers to achieve robust outcomes.

## 5 Self-reflection: writing as a process of inquiry

I, the second and corresponding author, subscribed to multi-paradigmatic research design and auto/ethnography as a research approach to explore the process of envisioning STEAM-based mathematics education during my PhD (Dahal, 2025a). The purpose of my inquiry was to develop the vision for STEAM-based mathematics education in Nepal. The vision seeks to integrate school and school community collaboration to foster an engaging pedagogical approach to mathematics education and/or classrooms, including school and school community involvement in developing transformative curriculum spaces, engaged pedagogy, transformative teacher professional development, and reflective teacher leaders. Likewise, given the purpose of my inquiry, the overarching question emerged for further exploration: How do contextual and universal perspectives contribute to envisioning transformative STEAM-based mathematics education in Nepal? These processes offered me the opportunity to uncover my personal values, beliefs, and practices. These processes of unpacking my personal values, beliefs, and practices largely rely on reading and writing, and their meaning is merely aligned with conscious writing. Conscious writing is a process of critical, reflective writing that allowed me to exercise my free will by articulating my research problems through writing narratives of my life worlds (Luitel, 2019). No doubt, in my inquiry, conscious reflective writing practice was also the process of expressing my feelings, belongings, doubts, and pleasures in the form of dialectical personal stories. These dialectical personal stories, in my inquiry, shaped my ways of thinking, being, valuing, and doing. In this regard, to capture the contextual and universal perspectives in my inquiry, I employed writing as a process of inquiry. Likewise, such an ontology-driven writing strengthened my inquiry through exposure to multiple research paradigms that allowed me to generate thick descriptions of myself and my research participants' personal-professional practices (i.e., interpretivism), to develop professional praxis through critical self-reflection (i.e., criticalism), and to utilize arts-based methods in making sense of and expressing unfolding subjectivities (i.e., postmodernism) (Luitel, 2019). In contrast, writing was not only a method of expressing ideas, but it also helped me in the meaning-making process, depending on the context.

The meaning-making process in auto/ethnographic inquiry is somewhat complex and challenges the ethos of conventional research's meaning-making process. In the process of challenging the ethos of conventional research's meaning-making process, the motto of my auto/ethnography inquiry was to explore anecdotal and personal experiences of self (insider) and others (culture) and connect this autobiographical story to wider cultural and social meanings and understandings to enrich the meaning-making process (Ellis et al., 2011). These processes required various layers of field texts (observations, interviews, memos, and notes, to name but a few) in the form of narratives. Narratives positioned me as the central character, considering relevant literature and viewing it through theoretical lenses. Ultimately, this process enabled me to generate evocative and contextual narratives, but it is unlikely to be enough to capture the broad spectrum of field data/texts. Because of this limitation, field data/texts were generated in various ways. Among these various ways of generating field texts, I employed dialectical, narrative, metaphorical, poetic, and visual logic and genres to make my different writing styles lively and viable through distinct logics and genres (Luitel, 2009).

The dialectical logic offered me the chance to create new spaces for envisioning STEAM-based mathematics education by promoting synergistic, complementary worldviews (integrative, holistic, and inclusive) (Bakhurst, 2008; Luitel, 2009). Moreover, it helped me balance contradictory perspectives and values through continuous practice, critiquing both others and myself in my inquiry. Likewise, in my inquiry, the narrative genre proved to be an important means for exploring multiple dimensions of life worlds (Luitel, 2019). The narrative genre further helped me to promote mythos-centric thinking that integrates place, people, action, and time in generating research texts rich in cultural-contextual knowing, being, and valuing that make the events understandable in the process of the inquiry (Taylor et al., 2012; Forchtner, 2020). Similarly, the metaphorical logic in my inquiry enabled me to envision STEAM-based mathematics education by capturing the complexity of the phenomenon to explore the meaning of concepts and ideas. As a result, this logic provided me with a platform for thinking and acting (Luitel, 2019) that made my writing meaningful and understandable. In addition to this, the poetic expressions in my inquiry provided me with a space for exploring imaginative aspects that were fundamental for understanding non-real, felt, mythical, perceptual, imagistic, and atypical realities. Finally, the visual genres in my inquiry helped me capture non-linguistic genres to represent phenomena under study by incorporating photographs, paintings, cartoons, collages, and creative models. I employed these genres to demonstrate the multi-vocal, embodied, and non-linear nature of knowledge claims. Through this process of perspectival and layered exploration, writing served as a means of inquiry, shaping both the quality of the meaning-making process.

## 6 Innovative data collection methods

The following section discusses the applications of innovative and emerging data collection methods such as *Pandheri Guff* and *Chautari Guff* .

### 6.1 Pandheri guff

*Pandheri Guff* is a culturally rooted Nepali method of collaborative inquiry that fosters open, reflective, and judgment-free dialogue—often among women scholars—to share lived experiences, co-create knowledge, and challenge dominant academic norms through storytelling and collective reflection (Dhungana et al., 2024). *Pandheri Guff* , literally meaning “talk in the light,” refers to early morning or evening conversations traditionally held by lamplight or firelight in Nepali communities (Dhungana et al., 2024). As a research method or data collection approach, this approach creates intimate conversational spaces for women, thereby encouraging multi-layered reflection, storytelling, and the sharing of personal experiences that might not emerge in formal interview settings. The temporal and spatial qualities of *Pandheri Guff* —occurring in the early morning or evening, often in domestic spaces, with soft lighting—create conditions for contemplative dialogue and emotional safety. This method has been valuable in research on traditional knowledge systems and intergenerational learning, where elders share stories and wisdom in culturally appropriate contexts (Dhungana et al., 2024). In this way, the collective nature of *Pandheri Guff* allows multiple perspectives to emerge within a single conversation. At the same time, the storytelling tradition embedded in this practice provides rich narrative data about cultural practices, values, and experiences. However, researchers must be sensitive to gender dynamics, family hierarchies, and the cultural protocols governing participation in these conversations.

### 6.2 Chautari guff

*Chautari Guff* is a long-standing Nepali tradition of open, informal public dialogue where scholars, activists, and community members gather to exchange ideas on development, democracy, and social issues in a participatory and reflective setting (Onta, 2023). Further, in traditional lens *Chautari Guff* refers to conversations held at traditional resting places (*Chautari*) along mountain paths, where travelers gather to rest, share news, and engage in community dialogue. As a data collection approach, this approach forces the natural convergence of diverse community members in neutral spaces that facilitate open communication across social boundaries. The public yet intimate nature of *Chautari* spaces creates unique opportunities to understand community dynamics, information flows, and collective problem-solving processes. These conversations often involve spontaneous participation from passersby, creating naturally occurring focus group discussions that reveal community perspectives on local issues and changes. Subscribing to the *Chautari Guff* in educational research has provided valuable insights into how communities understand and respond to educational innovations, as well as how traditional knowledge is shared and preserved where data is co-constructed. However, the method requires patience, cultural sensitivity, and willingness to engage with whoever happens to be present, rather than controlling participant selection.

## 7 Final remarks

This reflection on qualitative data collection methods, from foundational interviews to culturally situated innovations, features a central tenet of qualitative inquiry: the method that serves the question and honors the context. Our journey through interviews, observations, focus group discussions, and self-reflection reveals that rigorous qualitative research is not about rigidly applying a textbook technique, but rather about engaging in a thoughtful, adaptive human process of co-constructing knowledge. Each method carries its strengths and vulnerabilities. Interviews, when conducted with sensitivity, unlock the depth of personal experience, while observations provide a window into the unspoken dynamics of social life. Focus groups harness the power of collective dialogue, and self-reflection turns the researcher's gaze inward, acknowledging their constitutive role in the inquiry. The innovation and/or emerging approach, however, lies in mastering these established methods to creatively adapt and generate approaches that resonate with the cultural and epistemological worlds of the participants. The introduction of innovative data collection methods, such as *Kurakani, Pandheri Guff, and Chautari Guff* , is not merely an additive contribution to a methodological toolkit. It is an assertion of epistemic diversity. These approaches challenge the dominance of Western-derived, individualistic data collection models by foregrounding relationality, collective wisdom, and the cultural specificity of knowledge exchange. These methods of data generation demonstrate that the most authentic and rich data often emerge from spaces of trust, reciprocity, and informal dialogue, rather than from formal, transactional question-and-answer sessions. Table 1 illustrates the data collection methods in qualitative research.

**Table 1:** Data collection methods in qualitative research.

**Data collection method**	**Purpose/Focus**	**Key features**	**Strengths**	**Challenges/Ethical considerations**	**Contextual relevance**
Interviews and informal conversations	To elicit in-depth individual experiences, beliefs, values, and meanings	Semi-structured and unstructured, probing questions, dialogic and co-constructed	Rich, nuanced data, flexibility, rapport building, suitable across designs	Time-consuming, interviewer bias, ethical sensitivity, power dynamics	Widely used across narrative inquiry, phenomenology, case study, and grounded theory
Observations	To understand social practices, interactions, and unspoken dynamics	Participant/ non-participant roles, field notes, immersion	Contextual depth to capture tacit knowledge	Risk of “going native”, role negotiation, researcher positionality	Central to ethnography and community-based research
*Kurakani* (informal conversational inquiry)	To generate authentic, trust-based insights through everyday dialogue	Unstructured, casual conversations, no rigid tools	Builds trust, culturally resonant, reduces formality	Lack of structure, demands reflexivity and ethical care	Strongly aligned with Nepali/South Asian relational cultures
Focus group discussion	To explore collective meanings and social interaction around an issue	Moderated group discussion (6–12 participants), semi-structured	Captures group dynamics, economical, stimulates diverse views	Dominant voices, groupthink, moderator bias	Useful for educational policy, program evaluation, and community studies
Self-reflection	To examine the researcher's lived experience and meaning-making	Conscious, reflective, narrative, dialectical writing; auto/ethnography	Deep reflexivity, epistemic transparency, transformative learning	Subjectivity critiques, demands methodological rigor	Particularly relevant for transformative, multi-paradigmatic research
*Pandheri Guff*	To create safe, contemplative spaces for sharing lived experiences (often women)	Early morning/evening dialogues, storytelling, collective reflection	Reveals hidden narratives, emotional safety, and rich cultural data	Gender norms, household hierarchies, and cultural sensitivity	Feminist, indigenous, and women-centered qualitative inquiries
*Chautari Guff*	To capture public, participatory community dialogue	Open, informal discussions in communal spaces	Collective knowledge construction, diverse participation	Limited control over participants, requires patience	Community-based research, democracy, education, and development studies

Nonetheless, the choice and execution of a data collection method are among the most critical determinants of a study's rigor and impact. It is a decision that carries significant ethical weight, as it dictates whose voices are heard, how they are represented, and what forms of knowledge are legitimized. As researchers, we must therefore be methodologically bilingual—fluent in established techniques while also being inventive and respectful enough to embrace culturally grounded practices. We do more than just collect data; we engage in a meaningful, ethical, and transformative practice of understanding the complex tapestry of human experience by moving beyond a one-size-fits-all approach and thoughtfully selecting—or even crafting—methods that align with our philosophical stance and research context. Thus, we can conclude that the future of qualitative research lies in a continued commitment to methodological rigor, creativity, and inclusivity, particularly in data generation.

## Data Availability

The original contributions presented in the study are included in the article/supplementary material, further inquiries can be directed to the corresponding author.
